# Supplementary material for: m6Aminer: Predicting the m6Am Sites on mRNA by Fusing Multiple Sequence-Derived Features into a CatBoost-Based Classifier
Source: Int J Mol Sci. 2023 Apr 26;24(9):7878. doi: 10.3390/ijms24097878 (PMC10177809; doi:10.3390/ijms24097878)
Supplement: Supplementary file 1 [file ijms-24-07878-s001.zip › Supplementary_Table.pdf]

Supplementary Table S1. The performance of the CatBoost-based model using different combinations of feature subset on the training dataset.

Feature ranking	ACC	AUC	Sn	Sp	F1	MCC
50	0.826±0.003	0.903±0.003	0.795±0.003	0.858±0.006	0.821±0.003	0.654±0.007
100	0.830±0.004	0.907±0.002	0.796±0.003	0.864±0.007	0.824±0.004	0.662±0.008
150	0.832±0.003	0.911±0.002	0.803±0.003	0.861±0.006	0.827±0.003	0.665±0.007
200	0.832±0.004	0.912±0.002	0.804±0.004	0.860±0.006	0.827±0.004	0.665±0.007
250	0.832±0.004	0.912±0.002	0.804±0.004	0.861±0.006	0.827±0.004	0.666±0.008
300	0.832±0.002	0.912±0.002	0.804±0.003	0.861±0.005	0.827±0.002	0.666±0.005
350	0.832±0.003	0.913±0.002	0.804±0.003	0.860±0.005	0.827±0.003	0.666±0.006
400	0.833±0.002	0.913±0.002	0.804±0.002	0.862±0.004	0.828±0.002	0.667±0.004
450	0.834±0.003	0.913±0.002	0.805±0.003	0.863±0.005	0.829±0.003	0.669±0.006
500	0.834±0.003	0.913±0.002	0.804±0.003	0.863±0.005	0.828±0.003	0.668±0.007
550	0.834±0.003	0.913±0.002	0.805±0.003	0.863±0.006	0.829±0.003	0.669±0.007
600	0.834±0.002	0.912±0.002	0.805±0.002	0.863±0.005	0.829±0.002	0.669±0.005
650	0.834±0.003	0.912±0.002	0.805±0.003	0.863±0.005	0.829±0.003	0.669±0.006
700	0.834±0.003	0.912±0.002	0.805±0.002	0.863±0.004	0.829±0.002	0.669±0.005
750	0.833±0.003	0.912±0.002	0.805±0.003	0.862±0.004	0.828±0.003	0.668±0.006
800	0.833±0.003	0.913±0.003	0.804±0.003	0.862±0.005	0.828±0.003	0.668±0.007
850	0.833±0.002	0.912±0.002	0.804±0.003	0.862±0.004	0.828±0.002	0.668±0.005
900	0.834±0.003	0.912±0.003	0.804±0.002	0.863±0.005	0.828±0.002	0.668±0.005
950	0.833±0.003	0.912±0.002	0.804±0.003	0.862±0.005	0.828±0.003	0.668±0.006
1000	0.834±0.003	0.912±0.002	0.805±0.003	0.863±0.005	0.829±0.003	0.669±0.006
1050	0.834±0.003	0.913±0.003	0.805±0.003	0.863±0.005	0.829±0.003	0.669±0.007
1100	0.833±0.003	0.912±0.002	0.805±0.003	0.862±0.004	0.828±0.003	0.668±0.006
1120	0.834±0.003	0.913±0.002	0.805±0.003	0.863±0.004	0.829±0.003	0.670±0.006

Supplementary Table S2. The detailed hyperparameters of the CatBoost-based model.

Hyper-Parameters	Optimal Values
Iterations	2000
learning_rate	0.03
random_seed	0
bagging_temperature	1
Depth	9
leaf_estimation_method	'Newton'
od_type	'IncToDec'
task_type	'GPU'
sampling_frequency	'PerTree'
loss_function	'MultiClass'
bootstrap_type	'Bayesian'

Supplementary Table S3. The independent testing details of three models

Model	Sub-training dataset	ACC	AUC	Sn	Sp	F1	MCC
m6Aminer	1	0.634	0.755	0.881	0.388	0.707	0.309
	2	0.661	0.761	0.866	0.456	0.719	0.353
	3	0.653	0.755	0.872	0.434	0.715	0.341
	4	0.658	0.754	0.869	0.447	0.717	0.348
	5	0.645	0.744	0.869	0.422	0.710	0.325
	6	0.639	0.751	0.884	0.394	0.710	0.319
	7	0.655	0.751	0.872	0.438	0.716	0.343
	8	0.633	0.752	0.878	0.388	0.705	0.305
	9	0.650	0.764	0.872	0.428	0.714	0.335
	10	0.644	0.752	0.881	0.406	0.712	0.327
	Average	0.647	0.754	0.874	0.420	0.713	0.331
m6AmPred	1	0.637	0.748	0.897	0.378	0.712	0.322
	2	0.630	0.745	0.894	0.366	0.707	0.305
	3	0.611	0.739	0.875	0.347	0.692	0.261
	4	0.647	0.745	0.884	0.409	0.715	0.334
	5	0.620	0.733	0.881	0.359	0.699	0.282
	6	0.609	0.723	0.884	0.334	0.694	0.262
	7	0.627	0.737	0.881	0.372	0.702	0.294
	8	0.609	0.718	0.891	0.328	0.695	0.265
	9	0.631	0.750	0.906	0.356	0.711	0.314
	10	0.605	0.710	0.878	0.331	0.690	0.250
	Average	0.623	0.735	0.887	0.358	0.702	0.289
DLm6Am	1	0.639	0.736	0.866	0.412	0.706	0.312
	2	0.666	0.743	0.884	0.447	0.726	0.368
	3	0.637	0.717	0.869	0.406	0.706	0.310
	4	0.653	0.738	0.866	0.441	0.714	0.338
	5	0.628	0.714	0.878	0.378	0.703	0.296
	6	0.639	0.727	0.884	0.394	0.710	0.319
	7	0.655	0.731	0.891	0.419	0.721	0.351
	8	0.639	0.725	0.866	0.412	0.706	0.312
	9	0.652	0.741	0.866	0.438	0.713	0.335
	10	0.616	0.729	0.884	0.347	0.697	0.274
	Average	0.642	0.730	0.875	0.409	0.710	0.322
