# Supplementary material for: m6Aminer: Predicting the m6Am Sites on mRNA by Fusing Multiple Sequence-Derived Features into a CatBoost-Based Classifier
Source: Int J Mol Sci. 2023 Apr 26;24(9):7878. doi: 10.3390/ijms24097878 (PMC10177809; doi:10.3390/ijms24097878)
Supplement: Supplementary file 1 [file ijms-24-07878-s001.zip › Suppmentary Data.pdf]

# Suppmentary Data S1

The excel sheets show the importance score of 1,120 features.

The 1,120 features are ranked in descending order according to the importance score.

Ranking	Feature_name	Importance_score
1	DNM43	1
2	K-mer7	0.98982725
3	Ksnpf12	0.5898087
4	PseEIIP89	0.56920228
5	PseEIIP88	0.51900199
6	DNM44	0.51812469
7	SCPseTNC39	0.40661775
8	SCPseTNC27	0.39058084
9	SCPseTNC26	0.38815147
10	PseEIIP19	0.38642015
11	K-mer43	0.34179443
12	K-mer55	0.29969219
13	K-mer39	0.29548079
14	PseEIIP101	0.29035332
15	K-mer42	0.27805922
16	PseEIIP85	0.27171001
17	Ksnpf15	0.23021372
18	Ksnpf28	0.22400261
19	K-mer234	0.21350636
20	SCPseTNC23	0.20100889
21	PseKNC91	0.20063776
22	Ksnpf37	0.19845123
23	Ksnpf53	0.19786875
24	K-mer182	0.18177754
25	K-mer4	0.18177397
26	NCP53	0.18043921
27	NCP68	0.17804497
28	DNM4	0.17555217
29	DNM34	0.17497493
30	PseKNC73	0.17272922
31	Ksnpf50	0.17228665
32	K-mer10	0.16792951
33	DBE72	0.16389205
34	K-mer247	0.15729064
35	K-mer171	0.15519198
36	DBE74	0.15234531

37	K-mer186	0.14874718
38	K-mer235	0.14784853
39	Ksnpf34	0.14677302
40	Ksnpf63	0.14590192
41	NCP55	0.14544872
42	Ksnpf66	0.14294782
43	DBE75	0.13229972
44	PseKNC84	0.12823737
45	NCP59	0.12603876
46	DNM45	0.12444608
47	DBE77	0.12019967
48	Ksnpf2	0.11525583
49	Ksnpf5	0.10998906
50	Ksnpf31	0.10783405
51	Ksnpf32	0.1056472
52	NCP2	0.10268625
53	K-mer231	0.10240005
54	K-mer185	0.10159197
55	K-mer167	0.1007407
56	K-mer183	0.10028287
57	K-mer31	0.09927622
58	NCP60	0.09739384
59	K-mer5	0.09565569
60	K-mer170	0.09504539
61	K-mer187	0.08892817
62	Ksnpf65	0.08729197
63	NCP110	0.0866489
64	K-mer295	0.08587349
65	PseKNC79	0.08549961
66	SCPseTNC38	0.08456716
67	PseKNC78	0.08409746
68	Hash19	0.08289356
69	Ksnpf60	0.08236681
70	K-mer71	0.0808418
71	PseKNC7	0.08072285
72	Ksnpf69	0.08023562
73	Ksnpf76	0.07803018
74	SCPseTNC15	0.07685946
75	K-mer33	0.07625941
76	Hash20	0.07619013
77	NCP5	0.07508574

78	K-mer54	0.07499644
79	PseKNC75	0.07481532
80	PseEIIP59	0.07473753
81	PseEIIP58	0.07414179
82	Ksnpf48	0.07356901
83	PseEIIP100	0.07342651
84	PseKNC80	0.07341341
85	PseEIIP87	0.07330521
86	Ksnpf22	0.073079
87	PseKNC3	0.07247482
88	PseEIIP49	0.07187445
89	NCP56	0.07142413
90	Ksnpf33	0.07115088
91	PseKNC71	0.07016253
92	SCPseTNC55	0.07007375
93	PseEIIP43	0.0699153
94	SCPseTNC7	0.06991388
95	DNM13	0.06850757
96	K-mer44	0.06820624
97	SCPseTNC25	0.06741648
98	K-mer13	0.06551688
99	PseEIIP23	0.06547588
100	DNM37	0.06543017
101	PseEIIP104	0.06535021
102	Ksnpf9	0.06533775
103	Ksnpf38	0.06438221
104	Ksnpf16	0.06357822
105	DNM1	0.06242392
106	PseKNC155	0.06186859
107	Ksnpf21	0.06162068
108	Ksnpf27	0.06138535
109	NCP116	0.06130663
110	PseEIIP69	0.06111939
111	NCP80	0.06053927
112	PseEIIP74	0.06049989
113	Ksnpf79	0.05970137
114	DBE86	0.05909881
115	NCP57	0.05897046
116	PseKNC131	0.05835785
117	Ksnpf80	0.05704537
118	PseKNC135	0.05688554

119	PseKNC147	0.05683725
120	Ksnpf18	0.05599505
121	PseKNC107	0.05537591
122	Ksnpf59	0.055093
123	PseKNC103	0.05435486
124	DNM31	0.05415582
125	DBE73	0.05372428
126	PseKNC43	0.05295111
127	DBE71	0.05224512
128	PseEIIP98	0.05101307
129	NCP89	0.05077387
130	K-mer1	0.05061681
131	K-mer298	0.05058413
132	PseKNC39	0.05034277
133	NCP11	0.05017192
134	K-mer53	0.04986198
135	NCP64	0.04982335
136	SCPseTNC52	0.04929715
137	SCPseTNC42	0.04892154
138	NCP47	0.04876771
139	PseKNC85	0.04836724
140	PseKNC35	0.04834639
141	PseKNC74	0.04799374
142	Ksnpf44	0.04777255
143	PseEIIP53	0.0474951
144	K-mer6	0.0470106
145	DBE84	0.0466756
146	PseKNC151	0.04659009
147	PseKNC31	0.04588254
148	PseKNC163	0.04541475
149	NCP98	0.04530205
150	NCP26	0.04475872
151	DNM6	0.04472449
152	K-mer15	0.04425472
153	Ksnpf49	0.04424964
154	K-mer20	0.04406986
155	K-mer23	0.0439016
156	NCP29	0.04382204
157	SCPseTNC61	0.04370243
158	NCP77	0.04345641
159	K-mer35	0.04318987

160	SCPseTNC4	0.04293156
161	K-mer233	0.04231388
162	PseKNC23	0.04210257
163	NCP23	0.04207851
164	Ksnpf43	0.04201217
165	Ksnpf11	0.04187348
166	SCPseTNC37	0.04155999
167	K-mer41	0.04128808
168	PseKNC15	0.04112071
169	PseKNC143	0.04097817
170	Ksnpf64	0.04081709
171	NCP107	0.0404634
172	PseKNC119	0.04035441
173	PseEIIP86	0.0401047
174	NCP8	0.0400737
175	Ksnpf70	0.04005502
176	K-mer184	0.03989731
177	K-mer245	0.03989197
178	K-mer311	0.03987106
179	PseEIIP33	0.03889442
180	SCPseTNC13	0.03888186
181	K-mer117	0.03848103
182	K-mer68	0.03839826
183	SCPseTNC28	0.03830897
184	DNM20	0.03776147
185	PseEIIP22	0.03741905
186	Ksnpf75	0.03721593
187	PseEIIP47	0.03696909
188	SCPseTNC1	0.03688388
189	K-mer11	0.03679624
190	DNM14	0.03644641
191	Ksnpf8	0.03619484
192	Hash23	0.03615365
193	K-mer188	0.03613567
194	PseKNC87	0.03606956
195	PseKNC11	0.03599047
196	K-mer119	0.0358431
197	SCPseTNC43	0.0358155
198	NCP113	0.03555564
199	K-mer22	0.03517294
200	DNM36	0.03505842

201	PseEIIP36	0.03504453
202	PseKNC51	0.0347631
203	PseKNC96	0.03463383
204	NCP50	0.03461144
205	PseKNC123	0.03450018
206	PseEIIP77	0.03425038
207	Hash21	0.03417659
208	DNM38	0.03411357
209	PseEIIP82	0.03408708
210	PseEIIP39	0.0340535
211	NCP101	0.03390471
212	K-mer37	0.03366203
213	NCP38	0.0335038
214	K-mer58	0.03348696
215	NCP83	0.03340499
216	SCPseTNC19	0.03339825
217	K-mer228	0.03326107
218	K-mer309	0.03316665
219	PseEIIP50	0.03310657
220	PseKNC92	0.03298891
221	SCPseTNC20	0.03297968
222	Ksnpf19	0.03296779
223	SCPseTNC30	0.03290274
224	Ksnpf25	0.03289365
225	Hash18	0.03227419
226	NCP65	0.03226391
227	SCPseTNC59	0.03211487
228	K-mer155	0.03208809
229	Ksnpf6	0.03205787
230	PseKNC160	0.03193638
231	PseEIIP83	0.0318904
232	K-mer46	0.03187178
233	PseEIIP37	0.03176738
234	SCPseTNC14	0.03155171
235	SCPseTNC21	0.03144534
236	PseEIIP20	0.03143273
237	Ksnpf1	0.03136208
238	PseEIIP5	0.03135383
239	PseKNC52	0.03124927
240	K-mer199	0.03122149
241	K-mer96	0.03117774

242	NCP17	0.03103969
243	K-mer8	0.03099618
244	NCP92	0.03098384
245	Ksnpf51	0.03097054
246	PseKNC132	0.03095781
247	K-mer30	0.03091516
248	PseEIIP96	0.03089959
249	DNM19	0.03075245
250	Hash17	0.03068763
251	Ksnpf47	0.03058019
252	DNM35	0.03057175
253	Ksnpf20	0.03045881
254	K-mer47	0.03044009
255	Ksnpf55	0.03032449
256	DNM32	0.03025249
257	PseEIIP52	0.0302427
258	DNM47	0.03020475
259	SCPseTNC17	0.03008717
260	PseEIIP18	0.03005841
261	NCP66	0.02999607
262	SCPseTNC33	0.0298618
263	DNM39	0.02984813
264	PseEIIP73	0.02976234
265	SCPseTNC46	0.02976227
266	K-mer57	0.02969972
267	PseKNC136	0.02967322
268	K-mer101	0.0296505
269	Hash36	0.02964872
270	K-mer52	0.02964775
271	DNM5	0.02963988
272	NCP119	0.02961071
273	Hash10	0.02958936
274	Hash15	0.0295851
275	SCPseTNC53	0.02950668
276	PseEIIP11	0.02944746
277	K-mer299	0.02939015
278	DNM17	0.02915516
279	DNM48	0.02912844
280	PseKNC112	0.02910209
281	PseKNC120	0.02909354
282	SCPseTNC60	0.02905914

283	SCPseTNC31	0.02891187
284	DNM16	0.02890946
285	PseEIIP105	0.02880359
286	NCP94	0.02878369
287	K-mer16	0.02874314
288	PseKNC137	0.02873691
289	DNM2	0.02872889
290	PseEIIP48	0.02872005
291	PseKNC154	0.02870551
292	Ksnpf17	0.02869245
293	PseEIIP84	0.02866136
294	DNM22	0.0286074
295	Ksnpf57	0.02858666
296	K-mer165	0.02858617
297	NCP48	0.02856296
298	Ksnpf10	0.02856283
299	K-mer21	0.02853749
300	Hash11	0.02852037
301	K-mer104	0.02849022
302	PseKNC116	0.02847536
303	PseEIIP46	0.02846001
304	K-mer29	0.02845781
305	K-mer121	0.02842309
306	PseEIIP90	0.02840985
307	K-mer38	0.0284098
308	PseEIIP81	0.02839887
309	DNM21	0.02831603
310	PseKNC72	0.028272
311	K-mer154	0.02822716
312	K-mer175	0.02821963
313	DBE83	0.02821349
314	NCP32	0.0282119
315	PseKNC27	0.02817127
316	Ksnpf45	0.02815918
317	Hash22	0.0281579
318	PseKNC139	0.02813417
319	DNM28	0.02810429
320	PseKNC144	0.02810341
321	K-mer34	0.02809855
322	SCPseTNC57	0.02804268
323	K-mer76	0.02798699

324	SCPseTNC18	0.02798394
325	PseKNC115	0.02797973
326	NCP70	0.02797733
327	PseKNC64	0.0279715
328	PseEIIP66	0.02795527
329	PseKNC164	0.02793636
330	PseKNC90	0.0278785
331	K-mer74	0.02786316
332	K-mer26	0.02778249
333	Ksnpf54	0.02777927
334	Hash1	0.02777373
335	K-mer302	0.02776769
336	SCPseTNC49	0.02772669
337	Ksnpf29	0.02767905
338	K-mer24	0.02765284
339	PseEIIP3	0.02763114
340	DBE85	0.02759218
341	PseEIIP32	0.02758094
342	K-mer140	0.02753932
343	K-mer241	0.02750972
344	NCP122	0.02750444
345	Hash12	0.02749898
346	PseEIIP4	0.02743781
347	PseEIIP40	0.02739682
348	K-mer75	0.02739111
349	DNM46	0.02739071
350	SCPseTNC56	0.02737402
351	K-mer293	0.02734922
352	PseKNC44	0.02731312
353	K-mer59	0.0273126
354	PseKNC108	0.02731211
355	PseKNC95	0.02730039
356	SCPseTNC6	0.02728863
357	PseKNC32	0.02727436
358	PseEIIP72	0.02727224
359	PseKNC40	0.02726307
360	PseKNC60	0.02723167
361	K-mer3	0.0272306
362	NCP118	0.02722659
363	DNM7	0.02718992
364	Ksnpf61	0.02718083

365	PseKNC98	0.02716994
366	Ksnpf4	0.02714256
367	SCPseTNC54	0.02713201
368	SCPseTNC36	0.02712639
369	Hash28	0.02708746
370	Ksnpf67	0.0270729
371	Hash37	0.02706599
372	DBE89	0.0270277
373	PseEIIP102	0.02695871
374	PseEIIP91	0.02691835
375	PseEIIP76	0.02687727
376	DBE140	0.02686969
377	PseEIIP57	0.02680829
378	K-mer310	0.02680735
379	PseKNC124	0.02679875
380	K-mer225	0.0267963
381	DBE59	0.0267932
382	Hash16	0.02678129
383	SCPseTNC10	0.02677677
384	PseKNC133	0.02675904
385	K-mer181	0.02674483
386	Ksnpf7	0.02666735
387	DNM23	0.02661487
388	PseEIIP93	0.02659347
389	DNM29	0.02656868
390	DNM8	0.02651007
391	Ksnpf35	0.02650788
392	PseEIIP12	0.02650643
393	SCPseTNC35	0.02649829
394	DBE69	0.02649148
395	PseEIIP2	0.02647423
396	Ksnpf42	0.02645377
397	SCPseTNC11	0.02642852
398	K-mer27	0.02640521
399	Hash8	0.0263764
400	K-mer40	0.02634598
401	PseKNC28	0.02634122
402	NCP117	0.02627813
403	Hash30	0.02627645
404	SCPseTNC41	0.0262609
405	PseEIIP28	0.02625437

406	SCPseTNC24	0.02616295
407	PseEIIP13	0.02614227
408	K-mer150	0.02613891
409	Hash9	0.02613291
410	K-mer84	0.02611946
411	K-mer9	0.0260685
412	PseEIIP27	0.02604385
413	PseEIIP44	0.02604177
414	DBE67	0.02600032
415	Ksnpf74	0.02598027
416	DBE142	0.02597217
417	K-mer152	0.02591796
418	K-mer333	0.02590466
419	K-mer36	0.02590231
420	DBE37	0.02587047
421	Ksnpf40	0.02584701
422	SCPseTNC40	0.02579605
423	Ksnpf30	0.02577183
424	K-mer12	0.02576543
425	PseKNC47	0.02576071
426	DBE156	0.0257234
427	PseKNC67	0.02571475
428	DNM24	0.0257058
429	NCP3	0.02569688
430	K-mer197	0.02569537
431	Ksnpf23	0.02569492
432	PseEIIP75	0.02567592
433	PseEIIP38	0.02565655
434	DNM33	0.02565061
435	SCPseTNC50	0.02562499
436	PseKNC140	0.02561331
437	DBE158	0.02558205
438	Ksnpf62	0.02557287
439	PseKNC16	0.02554963
440	PseKNC20	0.02552736
441	Ksnpf73	0.02552389
442	NCP71	0.02552106
443	Ksnpf68	0.02551038
444	DBE152	0.02547739
445	DBE87	0.02544033
446	Hash25	0.02543384

447	SCPseTNC2	0.02543235
448	Hash39	0.02542288
449	DNM26	0.02541703
450	K-mer290	0.02540643
451	PseKNC94	0.025396
452	NCP104	0.02538723
453	PseKNC145	0.02538327
454	K-mer168	0.02534729
455	DBE24	0.02531151
456	SCPseTNC5	0.02529378
457	DBE154	0.02529337
458	PseKNC68	0.02528409
459	PseKNC89	0.02526763
460	K-mer294	0.02526169
461	PseEIIP63	0.02525811
462	Hash35	0.02524006
463	PseKNC159	0.0252254
464	DBE155	0.02519545
465	DBE122	0.02519533
466	PseEIIP80	0.02518797
467	K-mer91	0.02517195
468	PseEIIP1	0.02516886
469	K-mer123	0.02515866
470	K-mer106	0.02513711
471	Hash34	0.02512617
472	PseKNC65	0.0251091
473	Hash38	0.02508305
474	K-mer314	0.02506145
475	K-mer147	0.02505613
476	K-mer72	0.02502581
477	DBE141	0.02500511
478	Hash29	0.02499878
479	DBE53	0.02499133
480	DBE64	0.02498609
481	Ksnpf36	0.02494071
482	PseEIIP51	0.02489001
483	Hash14	0.02488921
484	DBE44	0.02488213
485	DBE138	0.02487759
486	Ksnpf46	0.02486985
487	PseEIIP79	0.02484514

488	NCP100	0.02484481
489	DBE119	0.02484349
490	PseKNC19	0.02484077
491	K-mer176	0.02483862
492	DBE47	0.02483336
493	Ksnpf56	0.02482793
494	PseEIIP71	0.02480156
495	PseEIIP42	0.02478562
496	Hash27	0.02476434
497	NCP44	0.02474852
498	SCPseTNC32	0.02471062
499	PseEIIP56	0.02469997
500	NCP33	0.02469807
501	Ksnpf52	0.02467132
502	K-mer14	0.024665
503	PseKNC1	0.02463038
504	NCP69	0.02462814
505	K-mer230	0.02462147
506	DBE3	0.02462036
507	DBE23	0.02461985
508	K-mer158	0.02461683
509	DNM18	0.02461674
510	Ksnpf41	0.0245954
511	PseKNC121	0.02455927
512	DBE151	0.02455153
513	K-mer162	0.02453893
514	DBE135	0.02450836
515	DBE100	0.02450736
516	NCP54	0.02450114
517	PseKNC59	0.02449571
518	PseEIIP78	0.02448864
519	PseKNC86	0.02448733
520	PseEIIP68	0.02448349
521	K-mer28	0.02447998
522	PseEIIP97	0.02447567
523	PseKNC6	0.02446457
524	DBE121	0.02446433
525	DBE56	0.02445963
526	Hash24	0.02445501
527	PseKNC63	0.0244451
528	K-mer160	0.0244417

529	Hash13	0.02443091
530	K-mer50	0.0244154
531	K-mer113	0.02441299
532	NCP18	0.02439454
533	Ksnpf71	0.02438176
534	PseKNC76	0.02438153
535	DBE70	0.02437818
536	PseEIIP17	0.02435363
537	PseEIIP10	0.02435243
538	DNM42	0.0243346
539	DBE45	0.02433416
540	K-mer25	0.02432335
541	Hash40	0.02430015
542	DBE126	0.02426536
543	K-mer18	0.02425835
544	PseKNC13	0.02424896
545	PseEIIP55	0.02423223
546	DBE42	0.02422372
547	PseKNC156	0.02422162
548	K-mer32	0.02421745
549	PseEIIP30	0.02420054
550	DBE124	0.02418464
551	Ksnpf58	0.02417234
552	K-mer60	0.02416709
553	PseKNC110	0.0241568
554	K-mer159	0.024156
555	DBE31	0.02414722
556	Hash31	0.02413201
557	NCP7	0.02412727
558	PseEIIP25	0.02411597
559	SCPseTNC58	0.02410792
560	PseKNC25	0.02409981
561	NCP115	0.02409464
562	Hash3	0.02406829
563	SCPseTNC22	0.0240172
564	PseKNC101	0.02401505
565	PseKNC97	0.0240129
566	NCP86	0.02401119
567	PseKNC70	0.02400258
568	NCP106	0.02399586
569	K-mer62	0.02398966

570	Ksnpf26	0.02398911
571	PseEIIP70	0.02398168
572	NCP20	0.02392062
573	PseKNC56	0.02392043
574	DBE108	0.02391284
575	PseKNC117	0.02391105
576	K-mer138	0.02389105
577	PseKNC126	0.02389006
578	PseKNC53	0.02389002
579	PseKNC122	0.02388463
580	NCP42	0.02388285
581	K-mer251	0.02387657
582	K-mer136	0.02386943
583	K-mer134	0.02384332
584	K-mer226	0.02382591
585	K-mer164	0.02382337
586	K-mer45	0.02376466
587	PseKNC36	0.02376103
588	PseKNC127	0.02375829
589	Hash2	0.02375427
590	K-mer200	0.02374704
591	PseKNC138	0.02373454
592	PseEIIP45	0.02373354
593	K-mer163	0.02372198
594	PseEIIP8	0.02372171
595	PseEIIP26	0.02370266
596	NCP15	0.02366749
597	DNM3	0.02366406
598	NCP85	0.02366216
599	DBE143	0.02366001
600	DBE92	0.02365287
601	PseKNC153	0.02363217
602	DBE46	0.02362044
603	DBE88	0.02361946
604	PseKNC10	0.02361546
605	PseKNC102	0.02361446
606	DBE21	0.0235972
607	PseKNC157	0.02358819
608	DNM10	0.02356856
609	PseKNC24	0.02356426
610	PseEIIP31	0.0235594

611	DBE10	0.02355715
612	NCP14	0.02355607
613	NCP93	0.02354952
614	DBE110	0.02354944
615	K-mer90	0.02353221
616	NCP103	0.02352766
617	Hash33	0.02351538
618	K-mer2	0.02351095
619	DBE52	0.02350837
620	PseKNC114	0.02348753
621	PseKNC2	0.02348308
622	DBE99	0.02347326
623	K-mer198	0.02345261
624	K-mer110	0.02345256
625	PseKNC105	0.02338665
626	DNM40	0.02337692
627	K-mer243	0.02336412
628	DBE48	0.02331242
629	K-mer238	0.02328883
630	Hash4	0.02328671
631	DBE95	0.02328522
632	DBE58	0.02325745
633	K-mer267	0.0232455
634	Ksnpf24	0.02322842
635	DBE68	0.02322792
636	DBE117	0.02322592
637	K-mer250	0.02321381
638	NCP46	0.02320947
639	DNM27	0.02319398
640	K-mer174	0.02319058
641	PseEIIP64	0.02318885
642	K-mer254	0.0231788
643	PseKNC18	0.02315901
644	DBE102	0.02315768
645	SCPseTNC16	0.0231574
646	DBE33	0.02312392
647	DBE12	0.02311668
648	NCP120	0.0230751
649	PseKNC113	0.02305832
650	PseKNC150	0.02305801
651	DBE159	0.02303292

652	PseEIIP29	0.0230279
653	K-mer315	0.02302561
654	NCP112	0.02302149
655	K-mer219	0.02301692
656	NCP67	0.02300636
657	NCP105	0.02299973
658	K-mer229	0.02299358
659	K-mer289	0.02295693
660	NCP123	0.0229547
661	DNM41	0.02295422
662	NCP74	0.02295298
663	K-mer122	0.02294818
664	NCP81	0.02294301
665	PseKNC152	0.02294267
666	K-mer85	0.02293305
667	K-mer111	0.02292976
668	PseKNC88	0.02292364
669	DBE36	0.02292261
670	DBE8	0.02290216
671	PseKNC77	0.02290098
672	PseKNC109	0.02288415
673	PseKNC21	0.02282967
674	PseKNC48	0.02282284
675	K-mer51	0.02280596
676	PseEIIP24	0.02279472
677	SCPseTNC64	0.02278612
678	K-mer316	0.02277093
679	DBE25	0.02274897
680	DBE54	0.02273674
681	K-mer201	0.02272398
682	PseKNC118	0.02271748
683	DBE120	0.02268929
684	PseKNC106	0.02268761
685	DBE109	0.02268099
686	Ksnpf77	0.02265875
687	K-mer244	0.0226563
688	NCP30	0.02264605
689	SCPseTNC12	0.02263933
690	DNM30	0.02263812
691	PseKNC158	0.02263354
692	NCP108	0.02261421

693	Hash6	0.02257421
694	PseKNC100	0.0225435
695	DBE4	0.02254284
696	PseKNC58	0.02254257
697	K-mer203	0.0225202
698	PseKNC29	0.02251593
699	NCP21	0.02249201
700	DBE114	0.02246938
701	PseKNC111	0.02246381
702	PseEIIP99	0.02245276
703	DBE123	0.02244605
704	Hash32	0.02244553
705	K-mer149	0.02242856
706	DBE144	0.02241697
707	DBE32	0.02240226
708	DBE14	0.0223976
709	K-mer19	0.02237118
710	DBE50	0.02233811
711	DBE112	0.02232806
712	K-mer204	0.02231636
713	K-mer325	0.0223113
714	PseKNC12	0.02230484
715	PseEIIP6	0.02229889
716	NCP96	0.02229829
717	K-mer153	0.02227648
718	DNM25	0.02227311
719	NCP78	0.02226111
720	DBE104	0.02225642
721	PseKNC128	0.0222411
722	DBE150	0.02222997
723	SCPseTNC47	0.02221815
724	K-mer206	0.02221411
725	DBE130	0.02220742
726	DNM9	0.02219934
727	K-mer70	0.02219262
728	K-mer17	0.02218799
729	DBE62	0.0221655
730	PseKNC9	0.02215183
731	PseKNC46	0.02213455
732	K-mer49	0.02213186
733	DBE66	0.02209535

734	DNM11	0.02209484
735	DBE91	0.02207945
736	NCP6	0.02207774
737	Ksnpf13	0.02204747
738	Hash7	0.02204264
739	PseKNC104	0.02203166
740	SCPseTNC9	0.02202743
741	NCP52	0.02202639
742	DNM12	0.02201921
743	DBE146	0.02201682
744	NCP9	0.02201442
745	DBE60	0.02201275
746	NCP37	0.02200583
747	K-mer48	0.02199629
748	SCPseTNC44	0.02199525
749	PseKNC93	0.02196395
750	PseKNC38	0.02195775
751	DBE113	0.02195424
752	Hash26	0.02194807
753	DBE90	0.02190942
754	DBE39	0.02190769
755	NCP95	0.02189207
756	DBE94	0.02186822
757	DBE129	0.02186537
758	PseKNC141	0.02181831
759	K-mer232	0.02180872
760	K-mer227	0.02178487
761	DBE136	0.02176246
762	PseEIIP9	0.02175889
763	DBE43	0.02175788
764	K-mer78	0.02175213
765	NCP35	0.02174369
766	K-mer107	0.02174326
767	K-mer79	0.02173648
768	DBE20	0.02173569
769	PseKNC14	0.02173466
770	PseKNC148	0.02172739
771	DBE35	0.02170925
772	K-mer80	0.0217057
773	K-mer65	0.02169404
774	K-mer211	0.02169028

775	NCP82	0.02167934
776	Ksnpf39	0.02165164
777	DBE107	0.02164611
778	Ksnpf72	0.02163875
779	K-mer92	0.02163459
780	DBE103	0.02163355
781	PseEIIP65	0.02162455
782	SCPseTNC48	0.02160981
783	DBE134	0.02160622
784	NCP24	0.02158084
785	PseKNC55	0.02156391
786	K-mer102	0.02156228
787	DBE153	0.02154543
788	NCP34	0.02154208
789	NCP111	0.02149137
790	DBE41	0.02149124
791	NCP73	0.02147265
792	K-mer145	0.02146942
793	DBE18	0.02146693
794	NCP91	0.021445
795	PseKNC149	0.02144157
796	PseKNC134	0.02143831
797	Ksnpf14	0.02137829
798	K-mer262	0.02137378
799	NCP84	0.021362
800	DBE128	0.02135932
801	SCPseTNC3	0.02133189
802	NCP51	0.02132049
803	DBE147	0.02131807
804	DBE55	0.02130684
805	K-mer326	0.02130218
806	PseKNC66	0.0213016
807	K-mer118	0.02130028
808	K-mer69	0.02129113
809	DBE96	0.02128846
810	DBE160	0.0212861
811	NCP28	0.02126161
812	PseKNC99	0.02125416
813	DBE111	0.02124747
814	NCP41	0.02123324
815	PseKNC5	0.02122905

816	NCP4	0.02120483
817	NCP121	0.02118611
818	DBE63	0.02116724
819	DBE137	0.02116392
820	PseKNC146	0.02115261
821	NCP12	0.02113135
822	K-mer246	0.02112513
823	NCP10	0.02112246
824	K-mer239	0.02109883
825	NCP1	0.02109215
826	PseKNC57	0.02107744
827	NCP39	0.02107489
828	DBE9	0.0210487
829	PseEIIP60	0.02102471
830	K-mer194	0.02099445
831	K-mer166	0.02097288
832	PseEIIP16	0.02095937
833	NCP114	0.02092712
834	PseKNC45	0.02092162
835	PseKNC162	0.02091902
836	PseEIIP15	0.0209012
837	K-mer248	0.0209005
838	DBE65	0.02086738
839	Ksnpf78	0.02086336
840	K-mer146	0.02085769
841	PseEIIP34	0.02083149
842	PseKNC17	0.02082937
843	K-mer249	0.02081758
844	DBE40	0.02076647
845	DBE98	0.02074624
846	K-mer63	0.0206956
847	K-mer99	0.02068757
848	NCP27	0.02064514
849	PseEIIP14	0.02064324
850	K-mer215	0.02060098
851	NCP49	0.02058099
852	NCP75	0.02057992
853	K-mer56	0.02057252
854	PseKNC49	0.02055182
855	PseEIIP61	0.02054822
856	DBE116	0.02054249

857	SCPseTNC29	0.02053709
858	NCP22	0.02053324
859	PseKNC129	0.0205292
860	DBE34	0.0205007
861	PseKNC41	0.02049556
862	DBE157	0.02049476
863	SCPseTNC63	0.02049006
864	K-mer330	0.02048974
865	K-mer114	0.02047222
866	DBE101	0.02046524
867	DBE57	0.02046392
868	PseEIIP92	0.02046321
869	DBE139	0.02045607
870	K-mer208	0.02044582
871	K-mer98	0.02044016
872	DBE2	0.02043306
873	PseKNC50	0.02040975
874	K-mer100	0.02037521
875	NCP97	0.02036638
876	SCPseTNC34	0.02034457
877	K-mer202	0.02034247
878	K-mer303	0.02033344
879	K-mer190	0.02033297
880	K-mer64	0.02032943
881	PseKNC142	0.0203234
882	SCPseTNC8	0.02032032
883	K-mer139	0.02031222
884	NCP13	0.02029193
885	NCP76	0.02026914
886	Hash5	0.02019672
887	PseEIIP41	0.02017295
888	K-mer312	0.02016933
889	K-mer270	0.02013709
890	DBE27	0.02012824
891	NCP36	0.02011955
892	K-mer88	0.02006491
893	K-mer133	0.02006158
894	DBE93	0.0200585
895	K-mer318	0.02005562
896	DBE11	0.0200466
897	K-mer307	0.02003042

898	PseKNC161	0.02001972
899	PseKNC34	0.01999035
900	PseKNC30	0.01994396
901	DBE29	0.01993061
902	PseKNC130	0.01991973
903	Ksnpf3	0.01991081
904	K-mer161	0.01991031
905	PseEIIP54	0.01990876
906	PseEIIP67	0.01990298
907	K-mer266	0.01989887
908	PseEIIP35	0.01989651
909	K-mer292	0.01985154
910	PseKNC37	0.01983608
911	DBE38	0.01982864
912	DBE145	0.01981813
913	DBE5	0.01980709
914	PseEIIP103	0.01979091
915	K-mer67	0.01977124
916	SCPseTNC62	0.01976398
917	PseKNC42	0.01971577
918	K-mer334	0.01970781
919	DBE16	0.01964004
920	NCP19	0.01961837
921	DBE76	0.01959822
922	DNM15	0.01949605
923	K-mer156	0.01947955
924	DBE125	0.01944909
925	K-mer115	0.01939636
926	PseEIIP94	0.01937104
927	DBE51	0.01934303
928	K-mer301	0.01934243
929	K-mer81	0.01933883
930	K-mer313	0.0193124
931	DBE127	0.01923494
932	K-mer73	0.01922813
933	DBE26	0.01922293
934	K-mer217	0.01920167
935	NCP25	0.01918024
936	K-mer120	0.01917986
937	DBE118	0.01917558
938	NCP79	0.01914696

939	K-mer124	0.0191403
940	DBE133	0.0191337
941	NCP45	0.01911804
942	DBE17	0.01909505
943	NCP43	0.01907148
944	K-mer255	0.01906421
945	K-mer82	0.01901574
946	PseKNC8	0.01901572
947	PseKNC62	0.01900301
948	K-mer268	0.01899642
949	K-mer127	0.01897541
950	DBE15	0.01896973
951	K-mer214	0.01895382
952	DBE13	0.01894355
953	K-mer216	0.01894014
954	DBE49	0.01893937
955	DBE28	0.01893626
956	DBE7	0.018915
957	NCP72	0.0189079
958	K-mer261	0.01888565
959	DBE1	0.01887471
960	K-mer213	0.01884209
961	K-mer220	0.01880111
962	K-mer66	0.01878727
963	DBE6	0.0187788
964	PseEIIP7	0.01876675
965	K-mer252	0.01872669
966	SCPseTNC45	0.01868474
967	NCP109	0.01863533
968	PseKNC61	0.01860825
969	K-mer126	0.01859
970	DBE106	0.01858986
971	K-mer253	0.01857488
972	NCP31	0.01847488
973	DBE78	0.01844741
974	K-mer242	0.01832923
975	DBE105	0.01830638
976	PseKNC33	0.01830493
977	DBE19	0.01827029
978	DBE97	0.01823865
979	K-mer86	0.01822175

980	K-mer169	0.01821034
981	PseEIIP95	0.01814693
982	SCPseTNC51	0.01812911
983	K-mer317	0.01810519
984	DBE115	0.01810224
985	K-mer331	0.01809493
986	K-mer83	0.01804963
987	PseKNC26	0.01802664
988	NCP102	0.01798679
989	PseEIIP62	0.01795917
990	NCP87	0.01795167
991	DBE30	0.01792112
992	DBE148	0.01790046
993	DBE149	0.01788769
994	DBE22	0.01786775
995	K-mer137	0.01772044
996	PseKNC54	0.01768238
997	K-mer328	0.017665
998	NCP88	0.01764864
999	K-mer212	0.01752475
1000	NCP58	0.01751916
1001	DBE132	0.01751095
1002	K-mer218	0.01746685
1003	K-mer272	0.01740346
1004	K-mer151	0.0173747
1005	K-mer335	0.01733372
1006	K-mer308	0.01732905
1007	K-mer264	0.01730433
1008	K-mer112	0.01726618
1009	K-mer193	0.01726497
1010	NCP99	0.01726153
1011	DBE61	0.01725986
1012	K-mer223	0.01724743
1013	NCP40	0.01719481
1014	K-mer210	0.01718935
1015	PseKNC69	0.01712273
1016	K-mer306	0.01698244
1017	DBE131	0.01697295
1018	K-mer94	0.0168378
1019	K-mer77	0.01682344
1020	K-mer89	0.01676341

1021	K-mer236	0.01670573
1022	K-mer291	0.01670125
1023	PseKNC22	0.01666885
1024	K-mer172	0.01648743
1025	K-mer209	0.01648454
1026	K-mer221	0.01647162
1027	K-mer109	0.0164715
1028	K-mer191	0.01645686
1029	K-mer116	0.01636998
1030	K-mer278	0.01634512
1031	K-mer329	0.01629914
1032	K-mer240	0.0162805
1033	K-mer61	0.01622819
1034	K-mer142	0.01622735
1035	NCP16	0.01617346
1036	K-mer273	0.01616463
1037	K-mer257	0.01613823
1038	K-mer93	0.01612649
1039	K-mer296	0.01609571
1040	K-mer128	0.0160839
1041	K-mer177	0.01592812
1042	K-mer148	0.0158999
1043	K-mer304	0.01589068
1044	PseKNC125	0.01589002
1045	K-mer179	0.01572592
1046	K-mer222	0.01567693
1047	K-mer305	0.01550398
1048	K-mer283	0.0153996
1049	K-mer319	0.01534561
1050	K-mer282	0.01534517
1051	NCP90	0.01533416
1052	K-mer320	0.01531094
1053	K-mer173	0.01522899
1054	K-mer256	0.01520948
1055	K-mer95	0.0151789
1056	K-mer280	0.01511844
1057	K-mer192	0.01508526
1058	K-mer207	0.01501854
1059	K-mer157	0.01489518
1060	K-mer286	0.01469792
1061	K-mer237	0.01468709

1062	K-mer275	0.0146489
1063	K-mer178	0.01435207
1064	K-mer332	0.0143228
1065	K-mer143	0.01422928
1066	K-mer180	0.01420348
1067	K-mer205	0.01419228
1068	K-mer265	0.01413005
1069	K-mer224	0.01401019
1070	K-mer274	0.01393419
1071	K-mer322	0.01388385
1072	K-mer144	0.01384602
1073	K-mer327	0.01384592
1074	K-mer135	0.01381573
1075	K-mer260	0.01370997
1076	K-mer288	0.01368182
1077	K-mer263	0.01358575
1078	K-mer103	0.01340211
1079	K-mer281	0.01333979
1080	K-mer131	0.01331463
1081	K-mer97	0.01321797
1082	K-mer196	0.01319154
1083	K-mer287	0.01287917
1084	K-mer132	0.01261469
1085	K-mer336	0.01252928
1086	K-mer323	0.01248615
1087	K-mer277	0.01243254
1088	K-mer276	0.0124266
1089	K-mer130	0.01200871
1090	K-mer271	0.0119437
1091	K-mer258	0.01187743
1092	K-mer108	0.01175609
1093	K-mer259	0.01168438
1094	K-mer125	0.0112988
1095	K-mer141	0.01119912
1096	K-mer195	0.01053595
1097	K-mer87	0.01030359
1098	K-mer129	0.01027463
1099	K-mer300	0.01003874
1100	K-mer321	0.00987155
1101	K-mer189	0.00944163
1102	K-mer279	0.00893084

1103	K-mer284	0.00854699
1104	K-mer285	0.00796082
1105	K-mer105	0.007692
1106	K-mer324	0.00767323
1107	K-mer269	0.00757763
1108	K-mer297	0.00718013
1109	PseEIIP21	0
1110	DBE79	0
1111	DBE80	0
1112	DBE81	0
1113	DBE82	0
1114	NCP61	0
1115	NCP62	0
1116	NCP63	0
1117	PseKNC4	0
1118	PseKNC81	0
1119	PseKNC82	0
1120	PseKNC83	0
